# Milk production and lactation length in Ankole cattle and Ankole crossbreds in Rwanda

**DOI:** 10.1007/s11250-020-02311-9

**Published:** 2020-06-08

**Authors:** Maximillian Manzi, Lotta Rydhmer, Martin Ntawubizi, Claire D’Andre Hirwa, Callixte Karege, Erling Strandberg

**Affiliations:** 1 Swedish University of Agricultural Sciences (SLU), Uppsala, Sweden; 2 Rwanda Agricultural Board (RAB), Kigali, Rwanda; 3 University of Rwanda (UR), Butare, Rwanda

**Keywords:** Breed groups. Crossbreeding, Milk yield

## Abstract

This study assessed daily milk yield (DMY), 100-day (MY100), and 305-day (MY305) milk yield, and lactation length (LL) in purebred Ankole cattle and Ankole crossbreds, and the influence of environmental factors on these traits. Milk yield data were obtained for 865 cows and 1234 lactations and analyzed using a mixed linear model. The overall least squares mean of DMY, MY100, and MY305 across breed groups was 2.7 L (*N* = 1234, SD = 1.7), 262 L (*N* = 959, SD = 176), and 759 L (*N* = 448, SD = 439), respectively, while the average lactation length was 256 days (*N* = 960, SD = 122). All factors included (breed group, season and year of calving, and parity) were significant for yield traits, except season of calving for MY305. First-parity cows had the lowest milk production, and fourth-parity cows the highest. For all traits, pure Ankole cows had the lowest milk yield. Among the crossbreds, there was no significant difference between Ankole × Friesian, Ankole-Jersey mother × Sahiwal sire, and Ankole-Sahiwal mother × Jersey sire, or between Ankole × Sahiwal and Ankole-Sahiwal mother × Sahiwal sire. It was concluded that Ankole crosses with Friesian or Jersey can be beneficial, even under a management system of limited nutrition as in Rwanda.

## Introduction

Livestock breeding programs play an important role in expansion of the agricultural sector, through improving the productivity of individual animals. In contrast to purebreeding, crossbreeding by itself does not produce genetic progress, but has advantage of exploiting complementarity of traits and heterosis (Simm 1998). Given the large differences in production traits between temperate breeds and local breeds, crossbreeding seems to be a logical solution to quickly improve production in tropical environments. However, poor adaptation of crossbreds to harsh production environments and low socio-economic support have raised doubts about the sustainability of crossbreeding in some parts of Sub-Saharan Africa. On the other hand, where local conditions allow proper implementation, crossbreeding can create substantial increases in animal performance and in farmer income (Roschinsky et al. 2015).

The dairy sector in Rwanda mainly consists of crossbred dairy cattle, contributing the major share of dairy production. The most frequent crosses are Holstein Friesian, Jersey, and Sahiwal with Ankole. The crossbreeding program at three research stations has produced several crosses of exotic dairy breeds with Ankole cattle, but these dairy crossbreds have not been evaluated for milk yield and environmental factors affecting their performance. Major environmental factors that may affect performance are herd, year and season of calving, and management (Epaphras et al. 2004). Various animal-related factors may also affect milk production, such as breed, age at calving, stage of lactation, parity, and milking frequency (Johnson et al. 2002). The aim of this study was to compare daily milk yield, 100-day, and 305-day milk yield, and lactation length for purebred Ankole and Ankole crossbreds, while also accounting for environmental factors influencing these traits.

## Materials and methods

### Location

Data were taken from milk production records at the Rwandan livestock research stations Songa (2° 24′ S, 29° 46′ E), Rubona (2° 30′ S, 30° 25′ E), and Kinigi (01° 43′ S, 029° 54′ E), located at altitude 1600, 1425, and 2400 m a.s.l., respectively. The rainfall pattern in Rwanda is bimodal, with a short rain season (September–December, season SRS) and a long rain season (March–May, season LRS). Correspondingly, the long dry season (season LDS) and the short dry season (season SDS) occur between June and August and January to February, respectively. Mean annual rainfall (1998–2017) at the Songa, Rubona, and Kinigi station was 1087, 850, and 1650 mm, respectively, and mean annual temperature was 20.1, 25.3, and 16.0 °C, respectively.

### Management of animals

The study animals were raised entirely on natural pastures without supplementary feeding, except mineral licks given ad libitum. Only younger calves, calving cows, and sick cows were housed, while the others were left to move freely in paddocks around the clock. Water was provided twice daily, in group troughs. Weaning of calves was done at an age of 8–13 months in groups, based on several criteria: overall vigor, ability to survive without milk, marked reduction in daily milk yield of the cow, reluctance to suckle, and, in some cases, loss of mothering ability in the cow or strong hostility towards the herdsman. In the first week after calving, calves were allowed to suckle colostrum freely. Thereafter, partial milking was done, where the calf first suckled the dam for about a minute to stimulate milk let-down, followed by actual hand milking, after which the calf was given the opportunity to suckle residual milk. The calves were then separated from their dams for the rest of the day. Cows were culled based on old age (e.g., loss of teeth) or low fertility. Routine disease control measures were undertaken, including treatments against ectoparasites and endoparasites.

### Data collection and trait definition

**Fig. 1:**
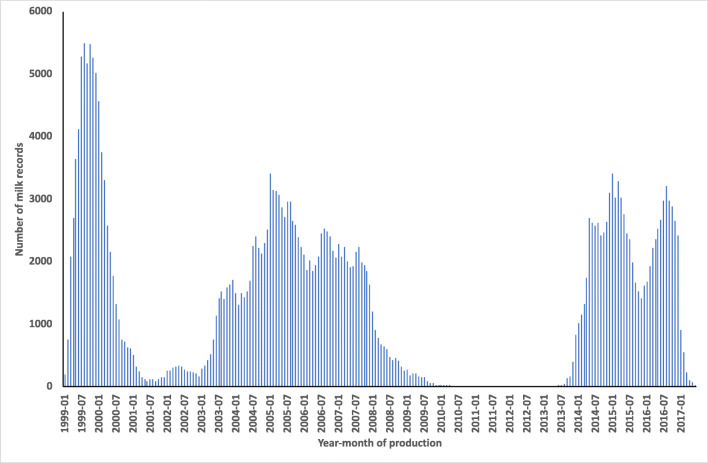
Frequency of daily milk yield records for various year-months

Milk yields were recorded daily at Songa station between 1999 and 2017 and at Rubona and Kinigi stations between 2013 and 2017. In total, information from 865 cows and 1234 lactations were available. The daily milk was collected in a bucket and measured with a graded jug. Yield was reported to the nearest 0.5 L. Daily milk yield cards were completed per cow and later computerized. Unfortunately, information was missing during certain periods (Fig. 1), most notably from 2010 through 2013, but also during 2001–2002. This had consequences for calculation of trait values.

Average daily milk yield (DMY) was calculated as the total lactation milk yield divided by the lactation length. For instance, if a cow had her first milk record at day 100 (due to a gap in recording) and her last milk record at day 200, the period was 101 days. Single days in a series that lacked milk yield values were allocated the average of the milk yield for the day before and the day after the gap. There were very few such gaps, and most identified were because the same date was given for 2 days in a row (for these, the dates were corrected).

Lactation yields, both early (100-day, MY100) and 305-day lactation yield (MY305) were calculated for cows that had their first day in milk (DIM) before day 28 postpartum and were producing milk until 100 or 305 DIM, respectively.

Lactation length (LL) was calculated only for those animals calving: (a) at least 500 days before 1 January 2001, (b) later than 1 January 2003, but at least 500 days before 1 January 2009, or (c) later than 1 January 2014, but at least 500 days before 1 July 2017. This was done to avoid gaps in the data recording that would result in too short LL values. To evaluate whether LL had an effect on DMY, the correlation between these traits was estimated using this dataset. It was not possible to estimate parametric lactation curves for individual cows using commonly used functions, owing to the large variation in lactation curve shapes.

### Data analysis

The fixed effects of breed group, season of calving, year of calving, and parity on all traits were analyzed using a mixed linear model in PROC Mixed of SAS (2012). A random effect of cow was included. Owing to the uneven distribution of data from the three stations over time and breeds, it was not possible to include a station effect. Two-way interactions between significant main effects were also tested. Breed groups included were pure Ankole (AA) and crossbreds with Friesian (F), Jersey (J), and Sahiwal (S). The breed groups were defined on the basis of mating system, e.g., AJ×S for a cow with an Ankole × Jersey crossbred mother and a Sahiwal father. The breed groups studied were AA, AF, AJ, AS, AJ×S, AS×J, and AS×S. Four season classes were studied: SDS, LRS, LDS, and SRS. Year of calving was classified into five groups: 1998–2000, 2001–2003, 2004–2006, 2007–2009, and 2014–2016. Parities were classified as 1, 2, 3, 4, 5+, and unknown. Owing to lack of birth dates for most animals, the assignation of parity was uncertain; what was designated as, e.g., parity 1 was actually first *known* parity, which may or may not have been the actual first parity, although the order of parities within cows was known from calving dates. Least squares means were calculated for each effect. Results were considered statistically significant at *p* < 0.05.

## Results

The overall average for DMY, MY100, and MY305 across breed groups was 2.7 L (*N* = 1234, SD = 1.7), 262 L (*N* = 959, SD = 176), and 759 L (*N* = 448, SD = 439), respectively, while the average LL was 256 days (*N* = 960, SD = 122). The correlation between LL and DMY was close to zero (− 0.08, *p* = 0.01). The variance of cow over total variance (repeatability) was 0.60, 0.48, 0.53, and 0.48 for DMY, MY100, MY305, and LL, respectively.

The least squares means of the fixed effects breed group, season, year of calving, and parity from a model without interactions are shown in Table 1. Breed group was significant for all yield traits, but not for LL. AA had the lowest milk yield. There was no significant difference in yield between AF, AJ×S, and AS×J, or between AS and AS×S.

**Table 1:** Number of observations (N), least squares means (LSM), and standard error (SE) for daily milk yield (DMY) and milk yield at 100 and 305 days of lactation (MY100, MY305), all in liters, and for lactation length (LL, days) for breed group, season, year of calving, and parity from a model without interaction effects

	DMY			MY100			MY305			LL		
Factor	*N*	LSM	SE	*N*	LSM	SE	*N*	LSM	SE	*N*	LSM	SE
Breed group												
AA	597	1.8^a^	0.1	497	171^a^	8	259	535^a^	27	477	253^a^	9
AF	138	4.6^b^	0.1	99	454^b^	17	34	1324^b^	54	100	254^a^	18
AJ	196	3.9^c^	0.1	162	397^c^	12	89	1157^c^	37	162	238^a^	13
AJ×S	65	4.5^b^	0.2	46	437^bc^	20	9	1500^b^	88	49	211^a^	21
AS	129	3.3^d^	0.1	94	314^d^	14	43	953^d^	47	119	227^a^	15
AS×J	65	4.5^b^	0.2	45	432^b^	20	9	1315^bc^	91	40	248^a^	23
AS×S	15	3.2^d^	0.3	16	318^d^	31	5	784^d^	116	13	258^a^	36
Calving season												
LDS	305	3.6^ab^	0.1	248	356^a^	10	93	1080^a^	40	254	228^a^	11
LRS	346	3.6^b^	0.1	282	356^a^	10	145	1066^a^	39	290	250^b^	11
SDS	199	3.8^a^	0.1	158	379^b^	12	85	1101^a^	40	178	252^b^	13
SRS	355	3.7^a^	0.1	271	351^a^	10	125	1077^a^	37	238	236^ab^	12
Year of calving												
1998–2000	290	3.7^ab^	0.1	237	345^ad^	16	111	1078^ad^	57	211	263^a^	18
2001–2003	91	3.8^b^	0.1	79	391^b^	15	44	1120^ab^	52	73	268^a^	17
2004–2006	349	3.6^a^	0.1	284	357^a^	10	163	1023^d^	35	343	242^a^	11
2007–2009	123	3.3^c^	0.1	88	323^cd^	12	25	963^cd^	50	75	180^c^	15
2014–2016	352	4.0^b^	0.1	271	385^b^	9	105	1221^e^	33	258	254^a^	10
Parity												
1	649	3.2^d^	0.1	474	322^d^	7	213	978^d^	28	512	226^ad^	8
2	108	3.4^de^	0.1	93	325^def^	12	47	1026^de^	44	90	275^b^	15
3	104	3.6^ce^	0.1	97	361^c^	13	52	1028^df^	45	87	277^b^	15
4	86	3.9^b^	0.1	73	368^bc^	14	30	1171^bc^	52	58	235^ad^	17
5+	182	3.5^cd^	0.1	169	348^cf^	14	90	1085^cef^	48	154	248^ab^	16
Unknown	76	4.5^a^	0.2	53	438^a^	22	16	1198^ac^	79	59	186^d^	22

^1^*AA* pure Ankole, *AF* Ankole (50%) × Holstein Friesian (50%), *AJ* Ankole (50%) × Jersey (50%), *AS* Ankole (50%) × Sahiwal (50%), *AJ×S* Ankole (25%), Jersey (25%) × Sahiwal (50%), *ASxJ* Ankole (25%), Sahiwal (25%) × Jersey (50%), *ASS* Ankole (25%) × Sahiwal (75%);

*SDS* short dry season (Jan–Feb), LRS = long rainy season (Mar–May), *LDS* long dry season (Jun–Aug), *SRS* short rainy season (Sep–Dec)

^abcdef^Mean values within columns with different superscripts differ significantly (*P* < 0.05)

Both DMY and MY100 were significantly affected by calving season. Cows calving in SDS had the highest yield, however, not always significantly different from other seasons. Year of calving significantly affected all four traits. The general pattern was similar for all yield traits, but for DMY and MY305, the highest year group was 2014–2016, whereas for MY100 the highest was 2001–2003. Year group 2007–2009 tended to have the lowest yield.

Parity significantly influenced all four traits. In general, yield increased from first to fourth parity, followed by a decline in parity 5. Lactation length increased during the first three parities, but then decreased.

**Fig. 2:**
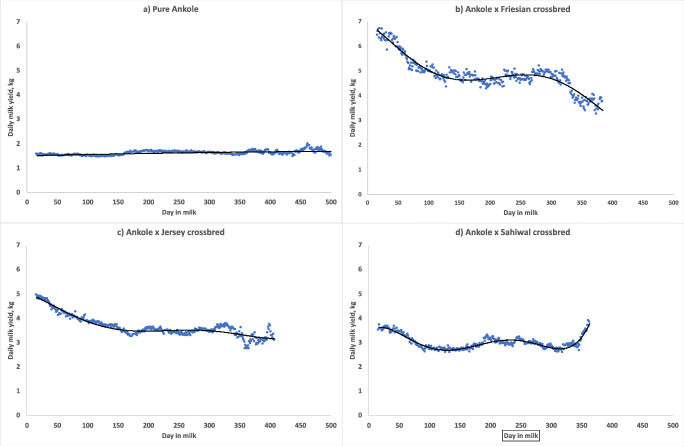
Lactation curves for **a** purebred Ankole (trendline equation: MY = 1.505 + 0.0006 d − 5.0 × 10^−7 ^d^2^); **b** Ankole × Holstein Friesian crossbreds (trendline equation: MY = 6.935 – 0.0152 d − 0.0002 d^2 ^– 2 × 10^−6^ d^3 ^– 6 × 10^−9^ d^4^ + 6 × 10^−12^ d^5^); **c** Ankole x Jersey crossbreds (trendline equation: MY = 5.037 + 0.0093 d − 1 × 10^–4^ d^2^ + 1 × 10^−6^ d^3 ^– 3 × 10^−9^ d^4^ + 3 × 10^−12^ d^5^); and **d** Ankole × Sahiwal crossbreds (trendline equation: MY = 3.488 + 0.0181 d − 0.0006 d^2^ + 6 × 10^−6^ d^3 ^– 2 × 10^−8^ d^4^ + 2 × 10^−11^ d^5^), where *d* is days in milk. A minimum of 10 cows was required for an average to be plotted

Estimation of parametric lactation curves for individual cows, with the intention of extending lactations shorter than 305 days, was unsuccessful, mainly owing to the large variation in lactation curve shapes, However, when average daily yields were calculated for different breed groups (Fig. 2), it was found that AF and AJ showed a decrease from a higher value early in lactation to a plateau at around 5 or 3.5 kg, respectively. Pure Ankole maintained almost the same yield, of about 1.5–1.8 kg, over the whole lactation and some cows milked for longer than 500 days.

For all three yield traits, breed group interacted with year class and parity. All other interactions were non-significant (results not shown). Most breed groups followed the general time trend (rather high values in the first 2-year classes, a decrease until 2007–2009, and then an increase to the last time class). However, for pure Ankole, this pattern was very weak and there was hardly any change over time. As regards the effect of parity, most breed groups followed the general trend of increased production up to parity 4, with a drop thereafter. This was most clearly seen for DMY and was generally the case for MY100, but was less clear for MY305. However, for pure Ankole, there was hardly any change with parity in any of the yield traits.

## Discussion

Milk production during a specified period of lactation is often used as a performance indicator of dairy cows. A common measure is milk yield per lactation or per year, or average milk yield per day. Sometimes, also lactation length can be of interest (Wondifraw et al. 2013). Owing to gaps in our dataset, we had more observations for average daily milk yield than for the other traits studied.

### Milk yield and lactation length—breed group differences

The mean DMY (1.8 L) observed for AA cows was within the range of estimates reported by others, e.g., studies in Uganda found DMY of 1–2.5 L/cow for AA under similar management (extensive grazing on natural pasture with no supplementation) (Galukande 2010; Kugonza et al. 2011). The average DMY for AF (50% Friesian) cows in this study (4.6 L) was lower than the 5.6 L found in studies on selected farms in Uganda (Galukande 2010). In Ghana, Darfour-Oduro et al. (2010) found a small but significant difference between pure Sanga and Sanga × Friesian cross (1.1 vs 1.4 L) both kept in an agropastoral system. In a study in the Democratic Republic of Congo on the performance of Ankole crossbred cows with different proportions of Friesian, Kibwana et al. (2015) found DMY of 5.4, 5.5, and 4.8 L in groups with no supplementary feeding with 44%, 38%, and 25% Friesian genes, respectively, and DMY of 7.8, 7.0, and 5.8 L, for corresponding groups with supplementary feeding. Compared with the 4.5 L/day observed in this study for AJ×S and AS×J, daily milk yields of 5.1 and 4.8 L for breed groups S×JA (50–75% Sahiwal, 12.5% Ankole, 12.5–37.5% Jersey) and J×SA (62.5% Jersey, 25% Sahiwal, and 12.5% Ankole), respectively, were reported from Burundi (Hatungumukama et al. 2009).

The MY305 values obtained in here differed significantly between pure Ankole and crossbreds (Table 1). Darfour-Oduro et al. (2010) found MY305 for pure Sanga cows and Friesian-Sanga crossbreds raised under an agropastoral system in Ghana of 244 kg and 339 kg, respectively. This is lower than the average obtained in our study. In a literature review, MY305 of Holstein Friesian × Indian breed crosses, with dams feed-supplemented and hand-milked and calves bucket-fed, ranged from 1707 to 3027 kg, while MY305 of pure Sahiwal and Sahiwal crosses ranged from 1633 to 1894 kg (Poonam et al. 2016). The lower yields in our study are probably due to poor nutrition associated with lack of feed supplementation.

Both total milk yield per year and per lactation can be influenced by lactation length. In most situations, the commonly accepted lactation length is 305 days. However, in our data, some cows were not milked for the whole 305-day lactation because they went dry or their lactation was terminated for some other reason. On the other hand, some cows were milked for longer than 305 days. The average lactation length ranged between 211 and 258 days depending on breed group (Table 1), which corresponded well with the range reported for tropical breeds and crossbreds. For instance, in Ghana, Darfour-Oduro et al. (2010) found that purebred Sanga and Friesian-Sanga crossbred cows had LL of 164 and 201 days, respectively. In a review of dairy cattle production in Ethiopia, Metekia and Nezif (2017) found a range for LL of 276–325 days for Holstein Friesian × Zebu crossbred animals. For Ankole cattle in Uganda a lactation length of 255 days was found (Kugonza et al. 2011).

We could not use all calvings to calculate LL, because of the gaps in the data (Fig. 1). There were two main gaps. The first (partial) gap was from around 2001 to 2003. Another more complete gap began in 2009 and lasted almost until 2014. To allow for an LL of at least 500 days, we decided to set an opportunity time of 500 days before the beginning of these two gaps as well as before the end of recording in 2017.

We found a very weak correlation between DMY and LL (− 0.08). This suggests that the milk yield of breed groups was not associated with lactation length. Initially, we intended to extend lactations shorter than 305 days by estimating parametric lactations curves based on part lactations. However, owing to the large variation in the shape of lactation curves, which the commonly used functions were unable to accommodate, this was not successful.

Based on the average DMY for the breed groups (Fig. 2), the yield of AF and AJ decreased from early in lactation to a stable level of around 5 or 3.5 kg, respectively. For AF, the yield decreased even more after around 300 days. For both AF and AJ, almost no cows continued milking after 350–400 days. There was a small decline in the first 50–100 days also for AS, but later they reached a plateau of about 3 kg. Pure Ankole produced an almost constant but low yield (1.5–1.8 kg) over the whole lactation. There were some AA cows that milked for longer than 500 days, even though in the statistical analysis we did not find significant effects of breed group on LL. It should be remembered that Fig. 2 shows raw yield averages. Therefore, by definition, cows that no longer milk are not included in the average.

The milk yield presented in this study was the milk offtake, because calves were allowed to suckle. An upper limit of suckled milk can be estimated based on how fast the calves grow. A previous study of calves at the three research stations included in this study found that the average daily weight gain was 0.4 kg/day (Manzi et al. 2018). Based on Dove and Axelsen (1979), an estimated 10.8 kg milk is needed per kilogram of daily gain. To achieve a weight gain of 0.4 kg/day only on milk, a calf would thus need to consume 4.3 L milk. At weaning (330 days), the calf would have consumed 1419 L. This is equal to or much higher than the measured milk offtake in this study (Table 1). However, it is difficult to know how much of the weight gain in calves comes from the milk it consumes and how much comes from pasture. Moreover, the proportion from milk decreases with age of the calf. Therefore, the values calculated here can be considered an upper limit of milk consumption.

### Season of calving

Season of calving had significant influence on DMY and MY100 (Table 1). Darfour-Oduro et al. (2010) in Ghana reported significant (*p* < 0.05) effects of season of calving on DMY and non-significant effects on MY305 and LL, for Sanga × Holstein Friesian crosses, but in the same study season had a significant effect on all traits in Sanga cows. The assumption is that calving season affects the yield during that season and the period thereafter. The season effect for a trait that is measured over a long time, e.g., MY305, is therefore a combination of a succession of seasons over 10 months, not only the actual calving season. This might be why the effect was diluted for MY305, whereas it was significant for MY100 (and DMY, which also contains some short lactations).

### Year of calving

Differences in milk yield between years are usually attributed to changes in management, feed availability, and other environmental factors (Nyamushamba et al. 2014). Therefore, the higher milk yield observed in the period 2014–2016 compared with previous years indicates positive effects of management practices or feed availability in that period. An effect of year of calving on milk production in Jersey and Fleckvieh × Jersey cows in a pasture-based feeding system has also been reported by Goni et al. (2015) in Ghana. Darfour-Oduro et al. (2010) found that an effect of year of calving was an important source of variation in DMY, MY305, and LL.

### Parity

Parity significantly influenced all traits. The lowest milk yield and LL were obtained in first-parity cows, with an increase to parity 4 and parity 3, respectively (Table 1). Similarly, in studies in Ghana, Goni et al. (2015) reported peak milk yield for Jersey cows in parity 3, while Darfour-Oduro et al. (2010) observed an effect of parity on DMY for both Sanga and Sanga × Holstein Friesian crossbreds, with peaks observed in parities 3 and 2, respectively. Nyamushamba et al. (2014) in Zimbabwe reported a significant parity effect (*p* < 0.05) on milk yield in a Red Dane herd, where the milk yield increased gradually from parity 1 up to parity 4, with a decline in parity 5. In that study, milk yield was lowest in parity 5, whereas in the present study the lowest yield was observed in first-parity cows. The lower milk yield in early parities may be because the animals are still growing and therefore nutrients are channeled to both body growth and milk production (Nyamushamba et al. 2014). The decline in parity 5 could be due to gradual degeneration of udder tissue with increasing parity. Owing to the uncertainty regarding birth dates in the present study, the parity number assigned might be somewhat overestimated (i.e., later parity cows might have been assigned to parity 1). Nevertheless, the general trends seem to coincide well with those in other studies.

## Concluding remarks

Breed group effect was significant for all yield traits. The breed group AA differed from all crossbreds, with the lowest milk yield. Among the crossbreds, there was no significant difference between AF, AJ×S, and AS×J, or between AS and AS×S. The better performance of AF, AJ×S, and AS×J was perhaps due to the higher milk production potential of Friesian and Jersey, so that the crossbreds with Ankole performed well both due to heterosis and additive effects. From these results, we conclude that Ankole × Friesian and Ankole × Jersey crosses can be beneficial even under a management system with limited nutrition as in Rwanda. However, from a conservation point of view, one should take steps to ensure that the local Ankole breed can survive in its own right and for use as a source of genes important for harsh conditions.
